# Identification of Immune&Driver Molecular Subtypes Optimizes Immunotherapy Strategies for Gastric Cancer

**DOI:** 10.3390/ijms27020696

**Published:** 2026-01-09

**Authors:** Jing Gan, Bo Yang, Shuangshuang Wang, Hongbo Zhu, Manyi Xu, Yongle Xu, Xinrong Li, Wenbo Dong, Yusen Zhao, Mengmeng Liu, Wei Feng, Yujie Liu, Junjie Duan, Shangwei Ning, Hui Zhi

**Affiliations:** College of Bioinformatics Science and Technology, Harbin Medical University, Harbin 150081, China; 18245115622@163.com (J.G.);

**Keywords:** gastric cancer, multi-omics, genomic alterations, immune microenvironments, molecular subtypes, immunotherapy

## Abstract

Immunotherapy has become a promising treatment for gastric cancer. However, its effectiveness varies significantly across subtypes because of heterogeneous immune microenvironments and genomic alterations. Here, we established Immune&Driver molecular subtypes CS1 and CS2 by systematically integrating multi-omics data for immune-related and driver genes. CS1 was linked to a better prognosis, while CS2 represented a poorer prognostic phenotype. CS1 displayed enhanced genomic instability, marked by higher mutation frequency and chromosomal alterations. In contrast, CS2 exhibited higher immune activity, with a higher density of immune cell infiltration and increased expression of chemokines and immune checkpoint genes. Among FDA-approved anti-cancer agents included in a pan-cancer drug sensitivity prediction framework, CS1 was predicted to be more sensitive to conventional chemotherapeutic agents, whereas CS2 was predicted to be more responsive to immune-related agents. In melanoma datasets, a CS2-like transcriptomic pattern was associated with improved response to anti-PD-1 therapy, with the combination of anti-PD-1 and anti-CTLA-4 showing more favorable response patterns compared to anti-PD-1 monotherapy. Additionally, we developed an immunotherapy response prediction model using PCA-based logistic regression according to the transcriptional expression of CS biomarkers. The model was trained in melanoma immunotherapy cohorts and validated across independent melanoma datasets, and it further achieved a higher AUC in an external gastric cancer cohort treated with anti-PD-1 therapy. Collectively, this study highlights immune and genomic heterogeneity in gastric cancer and provides a hypothesis-generating framework for exploring immunotherapy response.

## 1. Introduction

Gastric carcinoma stands as the third leading cause of cancer-related mortality and the fifth most prevalent malignancy globally [1]. Although the treatment of gastric cancer has made significant advancements in recent years, the survival outcomes of patients have been enhanced through surgery, chemotherapy, molecular targeted therapy, radiotherapy, or combination therapy; the overall efficacy of these treatments remains limited [2]. Immunotherapy, an emerging therapeutic modality, has achieved significant efficacy across various cancer types [3]. It has also been recognized as one of the most promising treatment strategies for gastric cancer [4]. Numerous clinical trials of immunotherapy for gastric cancer have been conducted, further substantiating its potential as a future cornerstone of treatment [5].

As a highly heterogeneous disease, gastric cancer is characterized by epidemiologic and histopathologic features according to phenotypic and molecular levels [6]. The response to immunotherapy has exhibited significant variability across different molecular subtypes and patient subgroups in gastric cancer [7]. Therefore, robustly classifying gastric cancer into clinically and molecularly distinct subgroups could significantly enhance the precision of treatment decisions. Traditional classifications of gastric cancer are predominantly based on anatomical and histological features. The World Health Organization (WHO) classification has categorized tumors into papillary, tubular, mucinous (colloid), and poorly cohesive carcinomas. Lauren classification employs two main histological subtypes, containing intestinal and diffuse subtypes [8]. In recent years, advancements in molecular biology technologies have facilitated the growing application of molecular subtype analyses in gastric cancer. The Cancer Genome Atlas (TCGA) research consortium has classified gastric cancer into four distinct subtypes: microsatellite instability, Epstein–Barr virus-positive, chromosomal instability, and genomically stable. This classification more accurately reflects the underlying biological characteristics of gastric cancer and offers novel insights for personalized treatment strategies [9]. However, previous studies have highlighted that, while current histopathological classifications may influence decisions regarding endoscopy or surgery, they ignore the molecular heterogeneity of gastric cancer and differences in immune response, and remain insufficient to guide personalized therapeutic approaches for individual patients [10]. Therefore, the identification and characterization of novel immune molecular subtypes in gastric cancer are crucial for improving the predictive accuracy of immunotherapy response, informing individualized treatment plans, and ultimately improving patient prognosis.

In this study, we defined Immune&Driver molecular subtypes in gastric cancer by systematically integrating multi-omics data of immune-related genes and driver genes. To characterize these Immune&Driver subtypes, we investigated their heterogeneity in overall survival (OS), genomic alteration, and tumor immune microenvironment (TiME). We predicted the immunotherapy response, especially anti-CTLA-4 and anti-PD-1 therapies, among distinct subtypes. In addition, we reconstructed molecular subtypes in independent gastric cancer cohorts based on the specific upregulated biomarkers. The survival outcomes, as well as the predicted sensitivities to chemotherapeutic and immune-related agents among Immune&Driver subtypes, were validated using these external datasets. The relevance of response to anti-PD-1 and anti-CTLA-4 therapies among subtypes was predicted using melanoma datasets. Finally, we identified key biomarkers linked to immunotherapy efficacy and prognosis, and developed a PCA-based logistic regression model to investigate their predictive performance in cohorts treated with anti-PD-1 and anti-CTLA-4 therapies.

## 2. Results

### 2.1. Identification of Immune & Driver Molecular Subtypes in Gastric Cancer

**Figure 1:**
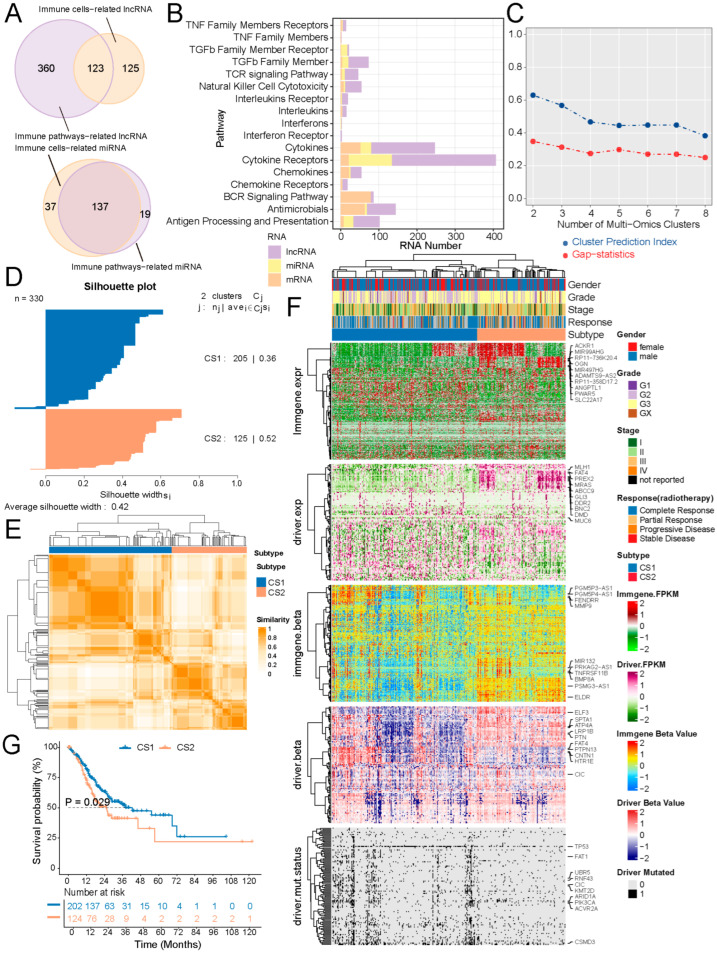
Identification of GC molecular subtypes. (**A**) Venn plot of immune-related lncRNAs and miRNAs. (**B**) Distribution of immune-related mRNAs, lncRNAs, and miRNAs. (**C**,**D**) Cluster prediction index, gap statistic, and Silhouette score for optimal subtype selection. (**E**,**F**) Consensus clustering and visualization of multi-omics data. (**G**) Kaplan–Meier curves showing OS differences between subtypes.

We identified 483 and 248 differentially expressed lncRNAs associated with 17 immune pathways and 50 immune cell types, respectively. By intersecting these two sets, a total of 123 immune-related lncRNAs were obtained (Figure 1A,B). Similarly, 137 immune-related miRNAs were identified by intersecting 174 and 156 differentially expressed miRNAs linked to 14 immune pathways and 45 immune cell types, respectively. 196 DEGs out of 1793 immune-related mRNAs were associated with 15 immune pathways.

We constructed an immune-related clustering model for STAD from the TCGA project using the MOVICS package by integrating multi-omics data of immune-related genes and driver genes. We determined the optimal cluster number to be two by evaluating the cluster performance using three clustering statistics (Figure 1C–E). Subsequently, integrative clustering analysis revealed two robust subtypes of gastric cancer, CS1 and CS2, distinguished by different molecular patterns, including transcriptome expression and DNA methylation patterns of immune-related genes and driver genes, as well as the mutation status of drivers (Figure 1F). The prognostic comparison analysis indicated that CS1 was significantly related to better OS than CS2 (Figure 1G). Additionally, although alternative clustering numbers at k = 3 and k = 4 were explored, neither yielded significant survival separation, and both were associated with reduced average silhouette widths compared with k = 2 (Figure S1), supporting k = 2 as the most robust and biologically relevant classification.

### 2.2. Prognostic Differences Between CS1 and CS2 Were Associated with Treatment Exposure and Established Clinicopathological Factors

In the TCGA cohort, treatment exposure and clinicopathological characteristics differed between the Immune&Driver molecular subtypes (Table 1). A higher proportion of CS1 patients were annotated as having received radiation therapy, additional radiation therapy, and additional pharmaceutical therapy. In contrast, CS2 tumors were characterized by more aggressive clinicopathological features, including a higher prevalence of high-grade disease (G3), advanced T stages (T3–T4), N stages (N3), M stages (M1), and overall pathological stages (stages III–IV). Furthermore, CS2 was significantly enriched for the genomically stable GS and EBV molecular subtype, whereas CS1 showed a higher representation of CIN and MSI subtypes.

**Figure 2:**
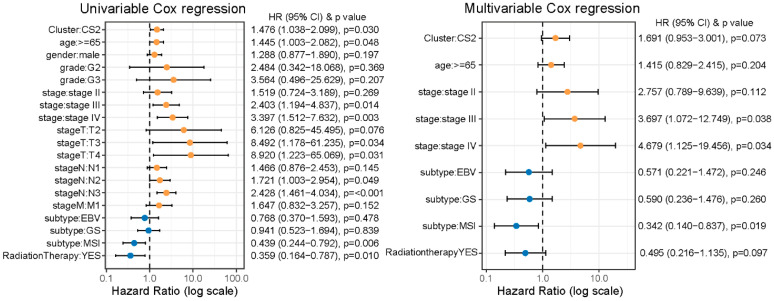
Cox regression analysis of Immune&Driver molecular subtypes in the TCGA cohort. Orange dots indicate HRs greater than 1, whereas blue dots indicate HRs less than 1.

In univariable Cox analyses, CS2 was related to notably worse overall survival compared with CS1 (HR = 1.48, 95% CI: 1.04–2.10; *p* = 0.03). However, after adjustment for established prognostic factors including age, tumor stage, treatment exposure, and TCGA molecular subtypes, the association between CS subtype and survival was attenuated, although the risk direction remained consistent (HR = 1.69, 95% CI: 0.95–3.00; *p* = 0.07) (Figure 2). These findings suggest that the survival difference observed between CS1 and CS2 was partly attributable to imbalances in known clinicopathological and treatment exposure across subtypes. Consistent with the multivariable Cox regression results, treatment-stratified analyses indicated that the relative survival trends between CS1 and CS2 were preserved in both radiotherapy and non-radiotherapy groups, although these differences did not reach statistical significance (Figure S2).

### 2.3. Cs1 Subtype Is Characterized by More Widespread Genomic Instability than Cs2

**Figure 3:**
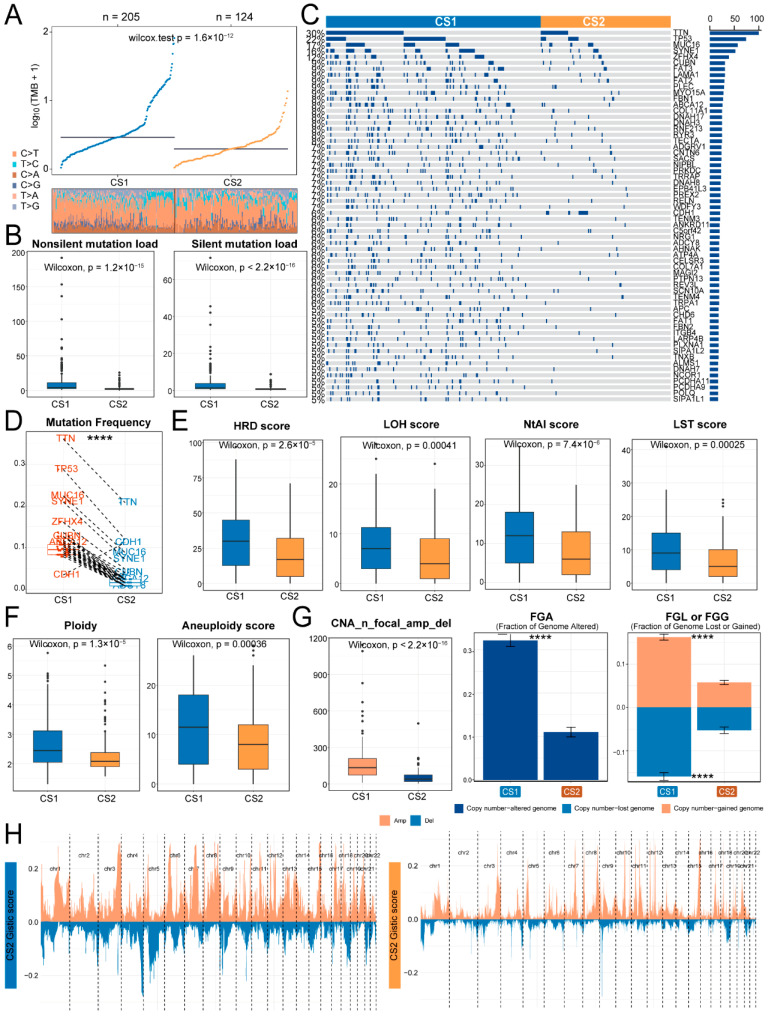
Genomic heterogeneity between subtypes. (**A**,**B**) TMB and mutation load comparison. (**C**) OncoPrint illustrating the somatic mutation landscape of differentially mutated genes between CS1 (*n* = 205) and CS2 (*n* = 124). Rows represent genes, and columns represent patients. The bar plot on the right indicates the number of samples harboring mutations in each gene, while the accompanying scale displays the mutation frequency of each gene across samples. (**D**) Paired-comparison boxplot for mutation frequency of differentially mutated genes. (**E**–**G**) Comparison boxplot of HRD, LOH, NtAI, LST, ploidy, aneuploidy, CNA, and fraction genome altered. (**H**) Comparison of SCNVs arm-level events. **** represents *p* < 0.0001.

To assess the genomic heterogeneity across Immune&Driver subtypes, we compared the TMB between CS1 and CS2 (Figure 3A). CS1 displayed a significantly higher TMB (median difference = 2.1, *p* = 1.6 × 10^−12^), and both non-silent and silent mutation loads were greater than CS2 (Figure 3B). To identify subtype-specific mutations, we compared the mutational frequencies of genes between CS1 and CS2. The mutation distributions of differentially mutated genes in patients were visualized using OncoPrint (Figure 3C). *TTN*, *TP53*, *MUC16*, *SYNE1*, *ZFHX4*, *CUBN*, *FAT3*, *LAMA1*, *FAT2*, and *PLEC* exhibited the highest mutation frequencies. Except for *CDH1*, all differentially mutated genes exhibited significantly higher mutation frequencies in CS1 (Figure 3D).

We subsequently compared genome instability between CS1 and CS2 subtypes by assessing various genomic alteration indicators. Our analysis demonstrated that loss of heterozygosity (LOH), number of telomere-extended allelic imbalance regions (NtAI), large-scale state transition (LST), and homologous recombination deficiency (HRD) scores were significantly higher in CS1 (Figure 3E). DNA ploidy and aneuploidy levels were notably increased in CS1 (Figure 3F). Additionally, CS1 demonstrated a greater number of copy number alterations (CNA) and a significantly higher fraction of genome altered (FGA) than CS2, with significantly greater specific gain (FGG) and loss (FGL) (Figure 3G,H). These findings indicate a more extensive disruption of the genomic landscape in CS1, characterized by both widespread genomic alterations and significant chromosomal aberrations.

### 2.4. Cs2 Subtype Demonstrates a More Dynamic and Resilient Immune Microenvironment

**Figure 4:**
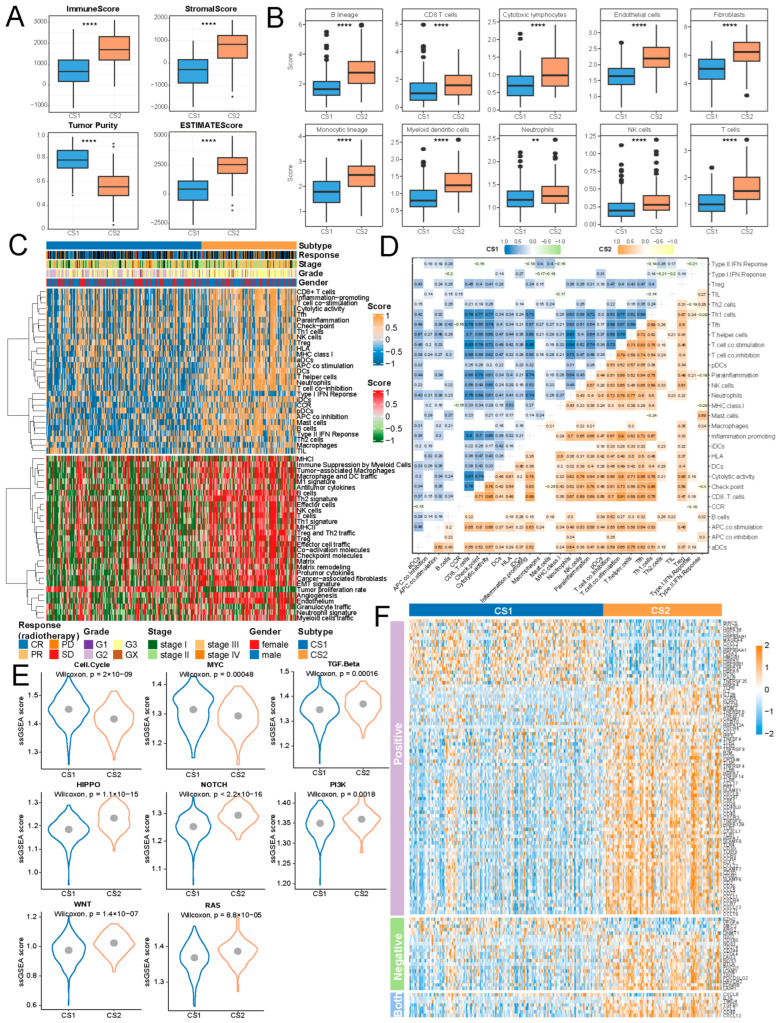
Immune landscape comparison between subtypes. (**A**,**B**) Comparison boxplot of Immune, stromal, ESTIMATE scores, tumor purity, and 10 immune cell infiltration. **** represents *p* < 0.0001; ** represents *p* < 0.01. (**C**) Activity score heatmap of classical immune signatures and immune-related Fges. (**D**) Correlations among classical immune signatures in subtypes. (**E**) Comparison of violin plots displaying the ssGSEA enrichment scores for 10 oncogenic pathways. (**F**) Expression heatmap of cancer immune cycle DEGs.

We conducted a comprehensive analysis comparing the TiME between CS1 and CS2 subtypes. Our findings revealed that scores of immune, stromal, and ESTIMATE were significantly higher in CS2 compared to CS1, along with significantly lower tumor purity (Figure 4A). CS2 also displayed significantly higher infiltrations of immune cells (Figure 4B), indicating a more prominent immune presence within its TiME. CS2 showed markedly higher enrichment levels for 29 classical immune signatures and 29 immune-related features compared to CS1 (Figure 4C). Furthermore, the correlation among classical immune signatures in CS2 was relatively higher than in CS1 (Figure 4D). These findings indicate that CS2 may possess a more active and robust immune landscape, potentially contributing to its distinct TiME.

The comparison of 10 oncogenic pathway scores between CS1 and CS2 revealed distinct pathway activation patterns (Figure 4E). Specifically, the cell cycle and MYC pathway were significantly activated in CS1, suggesting a heightened proliferative and oncogenic signaling activity [11,12]. In contrast, CS2 exhibited significant activation of Wnt, HIPPO, NOTCH, PI3K, RAS, and TGFβ pathways. The Wnt, HIPPO, and TGFβ pathways are closely linked to tumor immune evasion [13], tissue homeostasis and regeneration [14], and metastasis [15]. PI3K, RAS, and NOTCH pathways play crucial roles in tumor proliferation, migration, and survival [16,17,18], indicating a more complex and diverse oncogenic profile of CS2.

The cancer immune cycle comprises progressive events that must be started and continuously iterated to facilitate a practical anti-cancer immune response, ultimately leading to the eradication of tumor cells [19]. In this context, CS2 showed a significant overexpression of more genes that promote cancer immune cycle processes than CS1 (Figure 4F).

### 2.5. CS2 Is Associated with Predicted Sensitivity to Immune Checkpoint and Chemokine-Targeted Therapies

**Figure 5:**
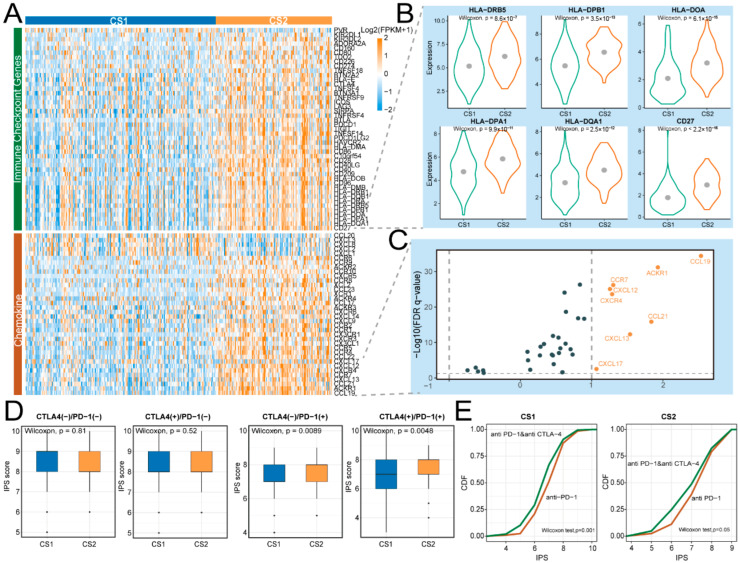
Response to chemokine and immune checkpoint therapy. (**A**) Expression heatmap of checkpoint genes and chemokines. (**B**,**C**) Immune checkpoint genes and chemokines significantly upregulated in the CS2 subtype (limma adj. *p* < 0.05, log2FC > 1). Grey dots represent genes with no significant differential expression. The dashed lines indicate the significance thresholds. (**D**) IPSs of patients from the TCIA database. (**E**) Cumulative distribution Function of IPSs comparing anti-CTLA-4 and anti-PD-1 combination therapy versus anti-PD-1 monotherapy across CS1 and CS2 subtypes.

Immunotherapy and chemotherapy represent the primary therapeutic approaches for advanced gastric cancer [20]. To determine potential strategies for immunotherapy, we compared the expression profiles of chemokine genes and immune checkpoint genes (ICGs) between Immune&Driver subtypes. Our results indicated that the expression levels of 45/79 ICGs and 36/63 chemokines were significantly different (limma, FDR < 0.05), and numerous ICGs and chemokines were overexpressed in CS2 (Figure 5A). Notably, ICGs, including *HLA-DRB5*, *HLA-DOA*, *HLA-DPB1*, *HLA-DQA1*, *HLA-DPA1*, and *CD27*, as well as chemokine genes such as *CXCL17*, *CXCL12*, *CXCR4*, *CCR7*, *CXCL13*, *CCL21*, *ACKR1*, and *CCL19*, were significantly up-regulated in CS2 (log2FC > 1) (Figure 5B,C). These findings suggest that patients classified as CS2 may exhibit a greater likelihood of benefiting from targeted immunotherapy strategies, particularly those ICGs- and chemokines-targeted therapies involving the above genes.

When comparing the IPS, we showed that the IPS of the ctla4_pos_pd1_neg group had no significant differences between CS1 and CS2 (Figure 5D). However, the IPS of the ctla4_neg_pd1_pos group was significantly higher in CS2, indicating that the CS2 cohort was more likely to respond to anti-PD-1 therapy rather than anti-CTLA-4, in contrast to CS1. Additionally, patients in CS1 and CS2 subtypes may exhibit a stronger response to combination therapy with anti-CTLA-4 and anti-PD-1 compared to treatment with anti-PD-1 alone (Figure 5E).

### 2.6. Cs1 Is Associated with Predicted Sensitivity to Chemotherapy Drugs in Gastric Cancer Cohorts

**Figure 6:**
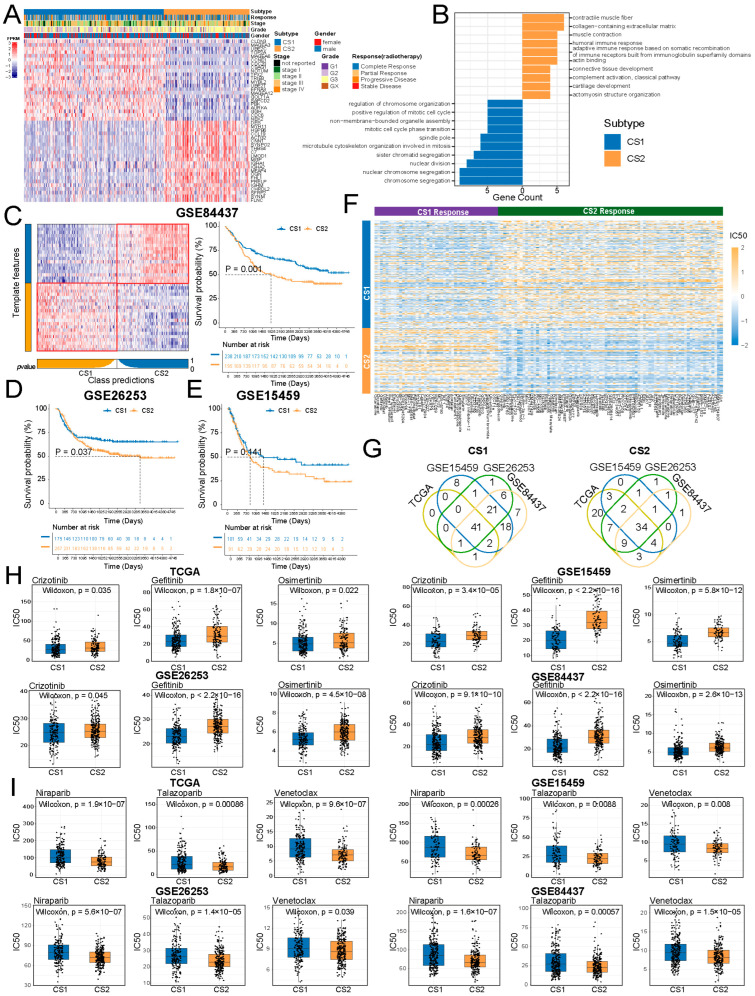
Drug response prediction across GC cohorts. (**A**,**B**) Expression Heatmap and functional annotation of the subtype-specific biomarkers in the TCGA cohort. (**C**–**E**) Subtype reconstruction and OS validation in external cohorts. (**F**) Drug response heatmap for CS1 and CS2 subtypes predicted by oncoPredict in TCGA. (**G**) Overlaps of response drugs for Immune&Driver subtypes across TCGA, GSE15459, GSE26253, and GSE84437 cohorts. (**H**,**I**) IC50 comparisons of FDA-approved chemotherapy drugs and immune-related drugs for CS1 and CS2 subtypes across cohorts.

To elucidate the distinct molecular features of CS1 and CS2, we identified 22 genes significantly upregulated in CS1 and 516 genes overexpressed in CS2. The 22 specifically upregulated genes in CS1 were considered candidate biomarkers for CS1. To ensure a balanced comparison and avoid bias from unequal feature sizes, we limited the number of biomarkers in CS2 to 22. Thus, the top 22 genes with the largest log2FC in CS2 were selected as potential biomarkers for CS2 (Figure 6A). To gain deeper insight into the biological significance of these subtype-specific biomarkers, we conducted functional annotation to investigate their roles in tumor biology (Figure 6B). For CS1, the biomarkers were primarily involved in cell cycle-related processes, including nuclear division, chromosome segregation, and the meiotic cell cycle [21,22,23], highlighting their potential role in promoting tumor cell proliferation [24]. CS2 biomarkers were linked to immune response-related biological processes, including humoral immune response, complement activation, and lymphocyte-mediated immunity [25,26,27]. These findings were consistent with our observations, where CS2 showed more immune cell infiltration, suggesting that the CS2 subtype may exhibit enhanced responsiveness to immune-related therapies.

We incorporated three additional gastric cancer cohorts and successfully reproduced the CS1 and CS2 subtypes using subtype-specific markers (Figure 6C–E). In line with observations from the TCGA cohort, patients in CS1 demonstrated better prognoses compared to CS2 across these datasets. We predicted the sensitivity of patients to 198 anticancer drugs and compared them between CS1 and CS2 (Wilcoxon test, FDR < 0.05, Figure 6F). We observed that patients in CS1 were predicted to exhibit sensitivity to 41 drugs, while CS2 patients were predicted to be sensitive to 34 drugs across the TCGA, GSE15459, GSE26253, and GSE84437 cohorts (Figure 6G). Crizotinib, gefitinib, and osimertinib are FDA-approved chemotherapy medications used to treat various cancer types and were predicted to be associated with increased drug sensitivity in CS1 patients (Figure 6H). In contrast, niraparib, talazoparib, and venetoclax are FDA-approved anticancer agents and were predicted to be associated with increased drug sensitivity in CS2 patients (Figure 6I). These drugs have been reported to modulate immune-related mechanisms across several cancer types. Specifically, niraparib has demonstrated synergistic antitumor activity in combination with PD-L1 blockade, primarily by enhancing the immune response by activating the cGAS/STING signaling pathway in ovarian cancer [28]. Tapsi et al. proposed that neoadjuvant talazoparib could change the TiME of breast cancer patients by increasing the infiltration of intratumoral and stromal T-cells [29]. Venetoclax, the first BCL-2 inhibitor approved by both the FDA and European Medicines Agency, has shown potential for preclinical combination with ICIs to enhance anticancer immunotherapy [30]. These findings underscore the distinct therapeutic efficacy of these agents in CS1 and CS2 subtypes, highlighting the potential of subtype-specific markers for guiding personalized treatment strategies in gastric cancer.

### 2.7. Sensitivity Differences Between Subtypes Increased When Comparing Anti-Pd-1 and Anti-Ctla-4 Combination Therapy to Anti-Pd-1 Monotherapy

**Figure 7:**
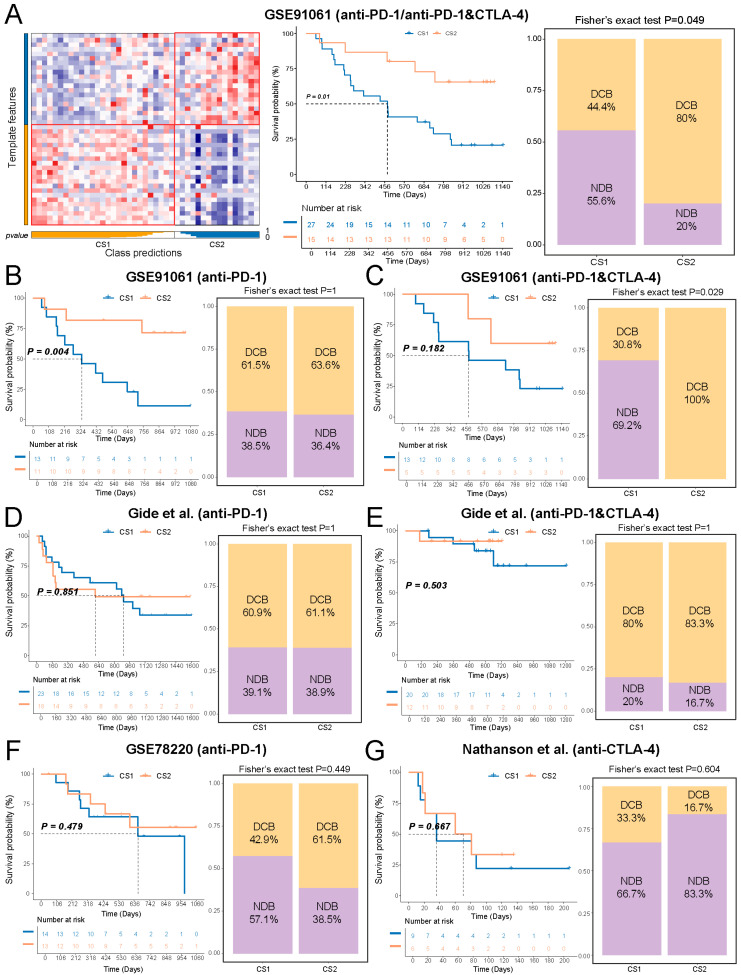
Immunotherapy response prediction for subtypes across melanoma cohorts. (**A**) Reconstruction of subtypes by NTP algorithm, OS differences between subtypes, and ICI response rates in the GSE91061 cohort. Kaplan–Meier curves and ICI response rates of molecular subtypes in the (**B**) anti-PD-1 therapy subgroup from the GSE91061 cohort, (**C**) anti-PD-1 and anti-CTLA-4 combination therapy subgroup from the GSE91061 cohort, (**D**) anti-PD-1 therapy subgroup from the Gide et al. cohort, (**E**) anti-PD-1 and anti-CTLA-4 combination therapy subgroup from the Gide et al. cohort, (**F**) GSE78220 cohort, and (**G**) Nathanson et al. cohort.

To explore the potential relevance of the Immune&Driver subtypes to immunotherapy response, we reproduced CS1- and CS2-like transcriptomic patterns in various melanoma datasets using subtype-specific markers. In the GSE91061 cohorts, patients exhibiting a CS2-like pattern showed significantly improved survival compared with CS1-like (Figure 7A). We observed an increased prevalence of DCB in patients with the CS2-like pattern relative to the CS1-like pattern.

When patients were stratified into receiving anti-PD-1 monotherapy and a combination of anti-PD-1 and anti-CTLA-4 therapy groups, the association between the CS2-like pattern and favorable survival outcomes was consistently observed (Figure 7B,C). Notably, while the proportion of DCB was comparable between CS1-like and CS2-like groups in the anti-PD-1 monotherapy cohort, patients in CS2-like groups demonstrated a complete response to the combination therapy. These observations were further corroborated by an independent melanoma dataset reported by Gide et al. (Figure 7D,E). Meanwhile, analysis of the GSE78220 and Nathanson et al. cohorts suggested that CS2-like patients were more likely to be responsive to anti-PD-1 rather than anti-CTLA-4 therapy, which was consistent with TICA results in the TCGA dataset (Figure 7F,G). These results indicate that, in melanoma cohorts, a CS2-like transcriptomic pattern is associated with more favorable outcomes following anti-PD-1 immunotherapy compared with CS1-like. Moreover, the immune program captured by the CS2-like pattern may be particularly relevant in the combination anti-PD-1 and anti-CTLA-4 treatment.

### 2.8. Development of Predictive Models for Ici Therapy Response by Cs Biomarkers

**Figure 8:**
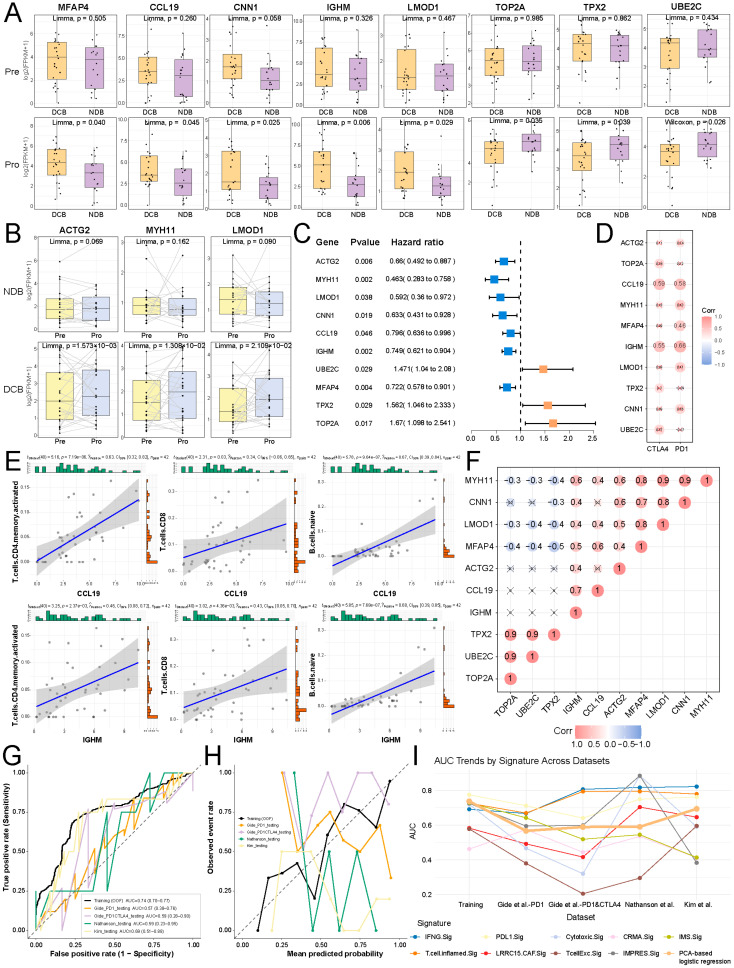
PCA-based logistic regression model for ICI response. Expression boxplot of feature biomarkers between (**A**) responders and non-responders prior to ICI treatment (top) and following ICI treatment (bottom), and (**B**) pre-treatment and post-treatment in non-responders (top) and responders (bottom). (**C**) Univariate Cox regression analysis for feature biomarkers. (**D**) Expression correlation between feature biomarkers and ICGs. (**E**) Expression correlation between *CCL19* and *IGHM* with the abundance of immune cells. (**F**) Expression correlation among feature biomarkers. (**G**) Receiver operating characteristic (ROC) curves in the training and independent testing cohorts, with corresponding AUC values. (**H**) Calibration plots of predicted versus observed response rates in the training (out-of-fold) and external cohorts. (**I**) AUC comparison of the PCA-based logistic regression model and other published models across training and testing cohorts.

To explore the transcriptional alterations of CS1 and CS2 biomarkers during ICI therapy, we examined transcriptomic profiles from paired ICI-treated melanoma patients in the GSE91061 cohort. Ten biomarkers exhibited significantly different expression levels between responders and non-responders after ICI treatment but not before treatment. Seven biomarkers demonstrated significant expression changes between pre- and post-treatment in responders, but not in non-responders. Among these, ten biomarkers associated with OS were utilized as CS feature biomarkers for predicting ICI therapy response (Figure 8A–C).

Further investigation of the potential relationship between CS biomarkers and ICGs revealed that *CCL19* and *IGHM* showed significant positive associations with *PD-1* and *CTLA-4* in expression (Figure 8D). Meanwhile, *CCL19* and *IGHM* also exhibited a significantly positive correlation with the infiltration of immune cells, including CD8^+^ T cells, activated CD4^+^ memory T cells, and naïve B cells (Figure 8E). Inhibition of PD-1 ligands markedly enhances the expansion and cytokine secretion capacity of memory or activated CD4^+^ T cells [31]. Immune checkpoint blockade (ICB) has transformed cancer therapy by unleashing CD8^+^ T-cell activity through targeting the suppression of *CTLA-4* and *PD-1/PD-L1* signaling pathways [32]. Additionally, *PD-1* has been identified across the principal human B-cell subsets obtained from peripheral blood and lymphoid tissues, especially on naive B cells [33]. Further integrative analyses indicated that most feature biomarkers were significantly positively associated with both CCL19 and IGHM (Figure 8F). These findings demonstrated that CS feature biomarkers, including *ACTG2*, *MYH11*, *LMOD1*, *CNN1*, *CCL19*, *IGHM*, *UBE2C*, *MFAP4*, *TPX2*, and *TOP2A*, exhibited synergistic interactions and were closely associated with *PD-1* and *CTLA-4*, which may act as crucial predictive indicators of ICI therapy efficacy.

Based on the above findings, we developed a predictive PCA-based logistic regression model and achieved an out-of-fold Area under the curve (AUC) of 0.74 in the training cohort (Figure 8G). In external validation, performance remained consistently above random, with AUCs of 0.57, 0.59, and 0.59 across the three melanoma cohorts, and a higher AUC of 0.69 in the gastric cancer cohort from Kim et al. Calibration showed reasonable agreement between predicted probabilities and observed outcomes in the training cohort, with increased variability in external datasets, particularly in smaller cohorts (Figure 8H). Compared with previously published models predicting immunotherapy response, the proposed model exhibited more balanced performance across cohorts without extreme degradation in any single dataset (Figure 8I). Confusion matrices summarizing sensitivity, specificity, and related metrics are provided in Table S2. Collectively, these results support the interpretation of the model as a hypothesis-generating predictor rather than a clinically optimized classifier.

## 3. Discussion

The advancement of high-throughput sequencing technologies has enabled the simultaneous acquisition of multiple genomic, transcriptomic, and DNA methylation data in gastric cancer. These multidimensional datasets offer deeper insight into the molecular characteristics of gastric cancer, facilitating its more precise classification. However, the combined contribution of immune-related and driver genes to the classification of gastric cancer subtypes remains to be explored.

We established a consensus classification using nine clustering algorithms integrating the heterogeneity of multiple omics from gastric cancer patients, including transcriptomic expression and DNA methylation of immune-related genes, as well as the transcriptomic expression, DNA methylation, and mutation status of driver genes. Our findings revealed two Immune&Driver molecular subtypes, CS1 and CS2. Patients with the CS1 subtype demonstrated more favorable clinical outcomes, while the CS2 subtype represented a poorer prognostic phenotype in patients. These two molecular phenotypes uncovered divergent genomic alterations and immune landscapes. Specifically, CS1 was characterized by greater genomic instability, marked by higher mutation rates and chromosomal alterations, suggesting a more aggressive tumor biology. In contrast, CS2 exhibited enhanced immune activity, with a higher density of immune cell infiltrates and elevated levels of immune checkpoint molecules, which may contribute to a stronger antitumor immune response. From a therapeutic perspective, CS1 was associated with conventional chemotherapy, while CS2 was associated with predicted sensitivity to immune-modulatory drugs and ICI therapies, particularly the combination of anti-CTLA4 and anti-PD-1 antibodies. These findings generate testable hypotheses regarding differential therapeutic vulnerabilities among molecular subtypes, which warrant further validation in prospective clinical studies.

To elucidate potential contributors to the observed survival differences, we compared the distributions of treatment modalities and clinical characteristics between CS1 and CS2. These clinicopathological disparities provide a plausible explanation for the observed survival disparity between CS1 and CS2. Although CS1 exhibited greater genomic instability and higher TMB, it was enriched for CIN and MSI subtypes and was more frequently receiving active therapeutic interventions, which may collectively contribute to improved clinical outcomes. In contrast, despite demonstrating increased immune cell infiltration and immune-related signatures, CS2 was associated with a predominance of GS tumors and more advanced disease at diagnosis, both of which are known to confer poorer prognosis in gastric cancer. The immune-active phenotype observed in CS2 may therefore reflect a compensatory or ineffective immune response within a more aggressive tumor context, rather than a protective anti-tumor immunity. Importantly, univariate Cox regression analyses identified age, molecular subtype, tumor stage, and treatment exposure as significant predictors of overall survival. After adjustment for these covariates in multivariate Cox proportional hazards models, the prognostic effect of CS subtype was attenuated, indicating that CS classification captures clinically relevant patterns of genomic instability and immune contexture that co-segregate with established molecular subtypes and treatment exposure, rather than representing a fully independent prognostic factor.

Before reconducting CS1 and CS2 subtypes in three independent gastric cancer cohorts, we extracted 22 genes with the largest log2FC from 516 significantly up-regulated genes in CS2 as a reduced CS2 biomarker panel. To assess the impact of feature reduction on subtype classification and biological characterization, we conducted comparative analyses using the full up-regulated gene set and the reduced biomarker panel. GO enrichment analyses demonstrated highly concordant enrichment of immune response–related biological processes between the two feature sets, indicating that the reduced biomarker panel retained the core functional characteristics of the CS2 subtype (Figure S3A). We further reconstructed the CS2 subtype in the above independent validation cohorts using the full CS2 feature set (Figure S3B,C). Unlike the results obtained with the reduced biomarker panel, the stratification did not yield significant differences in overall survival between CS1 and CS2 across these external datasets. These findings underscore the critical role of feature selection in achieving robust and generalizable molecular subtyping. Although the full CS2 feature set captures immune-related biological processes, its application in independent cohorts failed to consistently reproduce survival differences between CS1 and CS2. This inconsistency suggests that incorporating a large number of features may introduce noise or cohort-specific effects that compromise classification stability. Conversely, the reduced biomarker panel preserves the essential biological signals defining the CS2 subtype while enhancing robustness and reproducibility across datasets, supporting its value as a more interpretable and generalizable representation of subtype identity.

Given the current lack of publicly available gastric cancer cohorts treated with ICIs, the extrapolation of gastric cancer-derived subtype markers (CS1/CS2) to melanoma datasets was undertaken as a methodological proof-of-concept, rather than a direct biological or clinical translation. Melanoma, with abundant transcriptomic and ICI response data, provided a suitable model to evaluate whether the immune-related transcriptional programs underlying CS subtypes capture broadly predictive relevance for immunotherapy responsiveness. It is important to emphasize that melanoma and gastric cancer differ substantially in their oncogenic drivers, tumor microenvironment composition, and baseline immune contexture. Accordingly, our melanoma-based analysis does not imply biological equivalence between these tumor types, nor does it suggest immediate clinical applicability of CS1/CS2 for guiding immunotherapy in gastric cancer. The observation of CS2-like expression pattern in melanoma is associated with enhanced immune infiltration and improved ICI response, indirectly supporting the conceptual robustness of the immune signatures underlying our classification. Nevertheless, definitive evaluation of the predictive value of CS1/CS2 for immunotherapy response must rely on primary validation within gastric cancer ICI-treated cohorts. Such datasets will be essential to determine whether these immune-related molecular subtypes can inform clinical decision-making in gastric cancer.

Additionally, we identified ten CS biomarkers whose transcription changes during ICI therapy were strongly associated with patient prognosis. Among these, MYH11^+^ cancer-associated fibroblasts have been reported to promote tumor migration through interactions with macrophages and were linked to poor prognosis in colorectal cancer [34]. Overexpression of *LMOD1* has been shown to increase apoptosis and CD4^+^ T cells and activate oxidative stress in melanoma [35]. A unique functional subset of CCL19-expressing dendritic cells has been identified, showing favorable responses to anti-PD-1 therapy and displaying migratory and immunomodulatory phenotypes in triple-negative breast cancer [36]. Vitro experiments involving si-UBE2C cells demonstrated a reduction in the release of the cytokine TGF-β1, leading to a decrease in the Treg cell population within co-culture systems in renal clear cell carcinoma [37]. Further studies revealed that upregulation of *FOXF1*/*MFAP4* promoted anti-tumor immune responses through augmented dendritic cell and CD4^+^ T cell infiltration, facilitating crosstalk between lung adenocarcinoma and immune cells, and induction of multiple anti-tumor immune pathways [38]. Additionally, research has shown that increased *TPX2* expression enhanced human CD8^+^ T-cell-mediated antitumor activity and amplifies therapeutic responsiveness to *PD-1* inhibition in hepatocellular carcinoma patient-derived xenograft mouse models, irrespective of anti–PD-1 administration [39]. Mechanistic studies by Wu et al. highlighted that *TOP2A* played a pivotal role in immunotherapy efficacy and the formation of vascular mimicry–like structures in non-small cell lung cancer, mediated by increased Wnt3a and *PD-L1* expression [40].

Finally, we developed a parsimonious PCA-based logistic regression model to predict immunotherapy response using the transcriptional patterns of ten CS biomarkers. This approach summarizes correlated biomarker signals into a single composite score, reducing model complexity and mitigating overfitting in small cohorts. The model achieved an out-of-fold AUC of 0.74 in the training cohort and showed consistently non-random performance across external datasets, with AUCs of 0.57–0.59 in three melanoma cohorts and a higher AUC of 0.69 in the gastric cancer cohort. Calibration was acceptable in the training cohort but more variable in external validation, particularly in smaller datasets, consistent with cohort heterogeneity and sample-size limitations. Compared with previously published signatures, our model demonstrated more balanced performance across cohorts without extreme degradation in any single dataset. Overall, these results support the model as a hypothesis-generating predictor, and further validation in larger gastric cohorts will be essential to confirm generalizability.

## 4. Materials and Methods

### 4.1. Data Resources

Multi-omic data for gastric adenocarcinoma (STAD) from the TCGA project, including transcriptomic profiles, somatic mutation information, DNA methylation and copy number variation data, as well as clinical annotations, were obtained from the UCSC Xena database (https://xenabrowser.net/ (accessed on 5 March 2024)). In this study, 330 tumor samples comprising these multi-omic data and corresponding prognostic clinical variables were analyzed. 

Three independent gastric cancer cohorts, including GSE84437 (*n* = 300) [41], GSE26253 (*n* = 432) [42], and GSE15459 (*n* = 192) [43], were obtained from the Gene Expression Omnibus (GEO, http://www.ncbi.nlm.nih.gov/geo (accessed on 23 February 2025)) database and used as external validation sets. These cohorts provided comprehensive transcriptomic profiles together with corresponding survival information.

We collected four immunotherapy datasets for melanoma to assess the clinical outcomes of CS1 and CS2 subgroups following immune checkpoint inhibitor (ICI) treatment. GSE91061 includes 42 patients with information on expression and treatment response, in which 24 patients received anti-PD-1 therapy and 18 patients underwent combined PD-1 and CTLA-4 inhibition [44]. A dataset generated by Gide et al. comprises 41 patients who received *PD-1* blockade and 32 patients treated with combined *PD-1* and *CTLA-4* inhibition [45]. GSE78220 contains 27 patients undergoing anti-PD-1 treatment [46], while a dataset from Nathanson et al. consists of 15 patients receiving anti-CTLA-4 treatment [47].

### 4.2. Acquisition of Immune-Related Genes and Driver Genes in Gastric Cancer

1794 immune-related genes were obtained from the Immunology Database and Analysis Portal (ImmPort, https://www.immport.org/shared/genelists (accessed on 7 March 2024)), a curated immunology database that organizes genes into 17 immune categories according to distinct molecular functions [48]. Immune-related long non-coding RNAs (lncRNAs) and microRNAs (miRNAs) were downloaded from the ImmLnc database (http://bio-bigdata.hrbmu.edu.cn/ImmLnc/ (accessed on 7 March 2024)), referring to 17 immune pathways and 50 immune cell types, as well as differentially expressed information between normal and gastric adenocarcinoma tissues [49]. Additionally, 110 driver genes associated with gastric cancer were retrieved from the NCG database, which collects known and predicted cancer driver genes with detailed driving annotation information [50].

### 4.3. Integration and Clustering Analysis of Multi-Omics Data

The multi-omics data comprising log2(FPKM + 1) expression values, gene-level DNA methylation beta values of immune-related and driver genes, as well as a binary somatic mutation matrix (0/1) for driver genes from the TCGA-STAD cohort, were integrated. For methylation data, remaining missing values after probe-to-gene aggregation were replaced with zero to ensure a complete numeric matrix for integrative analysis. Uninformative features and samples (e.g., all-zero rows/columns) were removed. Because all omics data were derived from a single TCGA cohort, no additional cross-cohort batch correction was applied.

The integrated multi-omics dataset was then input into the ‘getClustNum’ function within the “MOVICS” R package (version 0.99.17) [51] to estimate the optimal number of clusters by jointly evaluating the Clustering Prediction Index (CPI) and gap statistic. Subsequently, integrative clustering analysis was performed via the ‘getMOIC’ method with a fixed random seed of 123. Nine clustering algorithms were applied, including SNF, PINSPlus, CIMLR, NEMO, LRAcluster, MoCluster, COCA, ConsensusClustering, and IntNMF. To enhance the reliability and stability of the clustering results, a consensus-based strategy was implemented in the ‘getConsensusMOIC’ function, which integrated the outcomes from multiple clustering algorithms. 

### 4.4. Analysis of the Genomic Instability

We separately derived tumor mutational burden (TMB) and fraction of genome altered (FGA) metrics using the ‘compTMB’ and ‘compFGA’ functions within “MOVICS”. The mutational frequencies of genes across different clusters were compared using the ‘compMut’ function. Additionally, the indicators of genomic instability were downloaded from GitHub (https://github.com/GerkeLab/TCGAhrd (accessed on 17 August 2024)) [52]. The GISTIC score of Somatic copy number variations (SCNVs) was derived for individual tumors using GISTIC 2.0 software.

### 4.5. Evaluation of the Immune Landscape

Immune infiltration metrics, including immune score, stromal score, ESTIMATE score, and tumor purity, were derived using the R package “estimate” (version 1.0.13) [53]. The immune cell infiltrations were evaluated using the “MCPcounter” package (version 1.2.0). 29 classical immune signatures and 29 immune-related functional gene expression signatures (Fges) were obtained from He et al. [54] and Bagaev et al. [55], respectively. For each sample, the enrichment of these signatures was calculated using ‘single-sample gene set enrichment analysis (ssGSEA)’ implemented in the “GSVA” R package (version 2.0.7) [56]. Oncogenic pathway definitions from Sanchez-Vega et al. [57], including ten pathways and 187 oncogenes, were used to compute sample-specific enrichment scores via ‘ssGSEA’.

### 4.6. Differential Expression and Functional Enrichment Analyses

The “limma” R package (version 3.62.2) was employed to conduct differential expression analysis [58]. Differentially expressed genes (DEGs) between Immune&Driver molecular subtypes were identified based on criteria of |log2FC| > 1 and FDR < 0.05. The biological functions of genes were annotated through Gene Ontology (GO) analysis implemented with the R package “clusterProfiler” (version 4.14.6) [59].

### 4.7. Validation of Molecular Subtypes

Subtype assignment for validation cohorts was performed using the Nearest Template Prediction (NTP) algorithm by calculating the similarity between samples and a predefined template. A key advantage of this algorithm is its model-free approach, which enables reliable category prediction for each sample using only biomarkers of known subtypes and expression of the test dataset [60]. The molecular subtypes of STAD were predicted in external validation datasets using the ‘runNTP’ function from “MOVICS”.

### 4.8. Prediction of Therapy Sensitivity

Immune phenotype score (IPS) for TCGA-STAD patients was obtained from The Cancer Immunome Atlas (TCIA) database to estimate the potential response to ICI therapy [61]. Drug sensitivity prediction was performed using the “oncoPredict” R package (version 1.2) [62]. The training resource was derived from the Genomics of Drug Sensitivity in Cancer2 (GDSC2) project, including 198 drugs after stringent quality control [63]. These compounds include conventional chemotherapeutic agents, targeted therapies, epigenetic modulators, and immune-related small-molecule inhibitors, reflecting a pan-cancer pharmacogenomic prediction framework rather than drugs restricted to gastric cancer. Predicted drug sensitivity (IC50) for each patient was generated using the ‘calcPhenotype’ function, with empirical Bayes batch correction enabled (batchCorrect = “eb”) to mitigate cross-dataset technical differences between the pan-cancer panel and gastric cancer. Recommended preprocessing options were applied, including power transformation of drug response values (powerTransformPhenotype = TRUE) and removal of low-variance genes (removeLowVaryingGenes = 0.2), to improve prediction stability. Drug annotations were retrieved from the DrugBank online database [64].

For melanoma datasets, patients were stratified according to immunotherapy response into durable clinical benefit (DCB) and no durable benefit (NDB) groups, with DCB defined by CR, PR, or SD accompanied by progression-free survival (PFS) > 6 months. NDB encompassed patients with progressive disease (PD), or SD with PFS ≤ 6 months [65].

### 4.9. Development of an Immunotherapy Response Prediction Model

We designated GSE91061 as the training cohort, and used the Gide et al. datasets together with the Nathanson et al. dataset as independent external testing cohorts in melanoma for the immunotherapy response prediction model. In addition, we incorporated a gastric cancer testing cohort derived from Kim et al., which included 45 patients treated with anti–PD-1 therapy [4]. The number of samples classified as DCB and NDB in the training set and the four external testing cohorts are summarized in Table S1.

To limit overfitting in a small-sample setting, we implemented a parsimonious predictive model based on logistic regression using a single composite predictor. Principal component analysis (PCA) was performed on ten CS biomarkers in the training cohort, and only the first principal component was retained a priori. PCA centering and loading parameters derived from the training data were fixed and applied unchanged to all validation and external cohorts.

Model performance in the training cohort was estimated using repeated 5-fold cross-validation with 20 repeats, and all training metrics were derived from out-of-fold predictions. Model discrimination was assessed using AUC, and calibration was evaluated using calibration plots and quantitative calibration metrics. External validation was performed in four independent cohorts that contained ten CS biomarkers. Furthermore, nine published signature models were collected to evaluate the predictive performance of the final model [66,67,68,69,70,71,72,73].

### 4.10. Statistical Analysis

R software (version 4.4.1) was used for all analyses in this study. Survival differences among molecular subtypes were evaluated by Kaplan–Meier curves whose significance was determined by the log-rank test. Pearson correlation coefficient was employed for correlation analysis. Univariable Cox regression analysis was conducted to assess the relationship between OS and the expression of CS biomarkers. Univariable and multivariable Cox regression analyses were performed to evaluate and adjust for the effects of treatment-related and clinicopathological variables on OS.

Significant differences in various analysis metrics between molecular subtypes were assessed by the Wilcoxon test. The significance of the DCB proportion difference between Immune&Driver subtypes was determined by Fisher’s exact test. These analyses consisted of a limited set of biologically pre-specified, hypothesis-driven two-group comparisons that were assessed independently, and, accordingly, no multiple testing correction was performed. For analyses involving multiple parallel comparisons, *p* values were corrected for multiple testing using the Benjamini–Hochberg procedure to control the false discovery rate (FDR). Specifically, differential expression analyses were performed using the “limma” framework with FDR correction, and comparisons of IC50 values across 198 anti-cancer drugs between subtypes were conducted using Wilcoxon tests followed by FDR adjustment.

## 5. Conclusions

Our study underscores the importance of integrating multi-omics data to define molecular subtypes for gastric cancer with distinct prognostic and biological characteristics. The identification of these subtypes provides insights into molecular heterogeneity and potential therapeutic relevance, highlighting how different treatment modalities may be associated with specific molecular profiles. Furthermore, subtype-specific biomarkers were leveraged as features to develop predictive models of immunotherapy response, serving as a methodological exploration of the immune programs captured by these subtypes. Collectively, these findings provide a hypothesis-generating framework for future studies and underscore the need for validation in gastric cancer cohorts with immunotherapy data before clinical translation.

## Figures and Tables

**Table 1:** Clinical characteristics of CS1 and CS2 in the TCGA cohort.

	Level	CS1	CS2
*n*		205	125
age_group (%)	<65	74 (37.2)	63 (51.2)
	≥65	125 (62.8)	60 (48.8)
gender (%)	female	76 (37.1)	37 (29.6)
	male	129 (62.9)	88 (70.4)
grade (%)	G1	4 (2.0)	4 (3.3)
	G2	96 (48.0)	25 (20.7)
	G3	100 (50.0)	92 (76.0)
stage (%)	stage I	35 (17.6)	8 (6.6)
	stage II	68 (34.2)	40 (33.1)
	stage III	79 (39.7)	61 (50.4)
	stage IV	17 (8.5)	12 (9.9)
stageM (%)	M0	184 (95.3)	109 (91.6)
	M1	9 (4.7)	10 (8.4)
stageN (%)	N0	65 (32.5)	35 (28.7)
	N1	57 (28.5)	29 (23.8)
	N2	43 (21.5)	26 (21.3)
	N3	35 (17.5)	32 (26.2)
stageT (%)	T1	15 (7.3)	3 (2.4)
	T2	47 (22.9)	17 (13.6)
	T3	91 (44.4)	65 (52.0)
	T4	52 (25.4)	40 (32.0)
TCGA subtype (%)	CIN	124 (67.8)	63 (54.3)
	EBV	11 (6.0)	15 (12.9)
	GS	5 (2.7)	32 (27.6)
	MSI	43 (23.5)	6 (5.2)
additional_pharmaceutical_therapy (%)	NO	14 (56.0)	23 (88.5)
	YES	11 (44.0)	3 (11.5)
radiation_therapy (%)	NO	132 (80.0)	85 (87.6)
	YES	33 (20.0)	12 (12.4)
history_of_neoadjuvant_treatment (%)	NO	205 (100.0)	125 (100.0)
additional_radiation_therapy (%)	NO	20 (83.3)	24 (96.0)
	YES	4 (16.7)	1 (4.0)

## Data Availability

The original data and code presented in the study are openly available on GitHub at: https://github.com/S-tian428/Immune-Driver-subtypes (accessed on 4 January 2026).

## Supplementary Materials

The following supporting information can be downloaded at: https://www.mdpi.com/article/10.3390/ijms27020696/s1.

## Abbreviations

WHO	World Health Organization
TCGA	The Cancer Genome Atlas
OS	Overall survival
TiME	Tumor immune microenvironment
STAD	Gastric adenocarcinoma
GEO	Gene Expression Omnibus
ICI	Immune checkpoint inhibitor
ImmPort	Immunology Database and Analysis Portal
lncRNAs	Long non-coding RNAs
miRNAs	microRNAs
CPI	Clustering Prediction Index
TMB	Tumor mutation burden
FGA	Genome altered
SCNVs	Somatic copy number variations
Fges	Functional gene expression signatures
SsGSEA	Single-sample gene set enrichment analysis
FDR	False discovery rate
DEGs	Differentially expressed genes
GO	Gene Ontology
NTP	Nearest Template Prediction
IPS	Immune phenotype score
TCIA	The Cancer Immunome Atlas
DCB	Durable clinical benefit
NDB	No durable benefit
CR	Complete response
PR	Partial response
SD	Stable disease
PFS	Progression-free survival
PD	Progressive disease
PCA	Principal component analysis
AUC	Area under the curve
ROC	Receiver operating characteristic
LOH	Loss of heterozygosity
NtAI	Number of telomere-extended allelic imbalance regions
LST	Large-scale state transition
HRD	Homologous recombination deficiency
CAN	Copy number alterations
FGA	Fraction of genome altered
FGG	Fraction of genome gain
FGL	Fraction of genome loss
ICGs	Immune checkpoint genes
ICB	Immune checkpoint blockade

## References

[1] Smyth E.C., Nilsson M., Grabsch H.I., van Grieken N.C., Lordick F. (2020). Gastric cancer. Lancet.

[2] Wang H., Ding Y., Chen Y., Jiang J., Chen Y., Lu J., Kong M., Mo F., Huang Y., Zhao W. (2021). A novel genomic classification system of gastric cancer via integrating multidimensional genomic characteristics. Gastric Cancer.

[3] Shi L., Chen S., Yang L., Li Y. (2013). The role of PD-1 and PD-L1 in T-cell immune suppression in patients with hematological malignancies. J. Hematol. Oncol..

[4] Kim S.T., Cristescu R., Bass A.J., Kim K.M., Odegaard J.I., Kim K., Liu X.Q., Sher X., Jung H., Lee M. (2018). Comprehensive molecular characterization of clinical responses to PD-1 inhibition in metastatic gastric cancer. Nat. Med..

[5] Högner A., Moehler M. (2022). Immunotherapy in Gastric Cancer. Curr. Oncol..

[6] Van Cutsem E., Sagaert X., Topal B., Haustermans K., Prenen H. (2016). Gastric cancer. Lancet.

[7] Refolo M.G., Lotesoriere C., Messa C., Caruso M.G., D’Alessandro R. (2020). Integrated immune gene expression signature and molecular classification in gastric cancer: New insights. J. Leukoc. Biol..

[8] Ma J., Shen H., Kapesa L., Zeng S. (2016). Lauren classification and individualized chemotherapy in gastric cancer. Oncol. Lett..

[9] The Cancer Genome Atlas Research Network (2014). Comprehensive molecular characterization of gastric adenocarcinoma. Nature.

[10] Chia N.Y., Tan P. (2016). Molecular classification of gastric cancer. Ann. Oncol..

[11] Fuentes-Antrás J., Bedard P.L., Cescon D.W. (2024). Seize the engine: Emerging cell cycle targets in breast cancer. Clin. Transl. Med..

[12] Park Y.R., Park S.M., Kim N., Jung J., Kim S., Kim K.I., Jang H.J. (2025). RNA-Binding Motif Protein 22 Induces Apoptosis via c-Myc Pathway in Colon Cancer Cells. Molecules.

[13] Luke J.J., Bao R., Sweis R.F., Spranger S., Gajewski T.F. (2019). WNT/β-catenin Pathway Activation Correlates with Immune Exclusion across Human Cancers. Clin. Cancer Res..

[14] Guo P., Wan S., Guan K.L. (2025). The Hippo pathway: Organ size control and beyond. Pharmacol. Rev..

[15] Zhao G., Zhao X., Liu Z., Wang B., Dong P., Watari H., Pfeffer L.M., Tigyi G., Zhang W., Yue J. (2025). Knockout or inhibition of DHPS suppresses ovarian tumor growth and metastasis by attenuating the TGFβ pathway. Sci. Rep..

[16] Tufail M., Hu J.J., Liang J., He C.Y., Wan W.D., Huang Y.Q., Jiang C.H., Wu H., Li N. (2024). Predictive, preventive, and personalized medicine in breast cancer: Targeting the PI3K pathway. J. Transl. Med..

[17] Ma H., Wu F., Bai Y., Wang T., Ma S., Guo L., Liu G., Leng G., Kong Y., Zhang Y. (2022). Licoricidin combats gastric cancer by targeting the ICMT/Ras pathway in vitro and in vivo. Front. Pharmacol..

[18] Liu L., Han F., Deng M., Han Q., Lai M., Zhang H. (2025). Crosstalk between GLTSCR1-deficient endothelial cells and tumour cells promotes colorectal cancer development by activating the Notch pathway. Cell Death Differ..

[19] Chen D.S., Mellman I. (2013). Oncology meets immunology: The cancer-immunity cycle. Immunity.

[20] Zhang C., Yin W., Yuan L.P., Xiao L.J., Yu J., Xiao W.M., Luo G., Deng M.M., Liu S., Lü M.H. (2024). Circadian rhythm genes contribute to the prognosis prediction and potential therapeutic target in gastric cancer. Sci. Rep..

[21] Yan X., Huang L., Liu L., Qin H., Song Z. (2018). Nuclear division cycle 80 promotes malignant progression and predicts clinical outcome in colorectal cancer. Cancer Med..

[22] Black E.M., Ramírez Parrado C.A., Trier I., Li W., Joo Y.K., Pichurin J., Liu Y., Kabeche L. (2024). Chk2 sustains PLK1 activity in mitosis to ensure proper chromosome segregation. Nat. Commun..

[23] Mensah L., Ferguson J.L., Shive H.R. (2019). Genotypic and Phenotypic Variables Affect Meiotic Cell Cycle Progression, Tumor Ploidy, and Cancer-Associated Mortality in a brca2-Mutant Zebrafish Model. J. Oncol..

[24] Glaviano A., Singh S.K., Lee E.H.C., Okina E., Lam H.Y., Carbone D., Reddy E.P., O’Connor M.J., Koff A., Singh G. (2025). Cell cycle dysregulation in cancer. Pharmacol. Rev..

[25] Barnes A.P., Khandelwal S., Sartoretto S., Myoung S., Francis S.J., Lee G.M., Rauova L., Cines D.B., Skare J.T., Booth C.E. (2022). Minimal role for the alternative pathway in complement activation by HIT immune complexes. J. Thromb. Haemost..

[26] Ferreiro-Iglesias A., McKay J.D., Brenner N., Virani S., Lesseur C., Gaborieau V., Ness A.R., Hung R.J., Liu G., Diergaarde B. (2021). Germline determinants of humoral immune response to HPV-16 protect against oropharyngeal cancer. Nat. Commun..

[27] Vanwijck R., Bouillenne C., Malek-Mansour S. (1975). Potentiation and arming of lymphocyte mediated immunity by sera from melanoma patients. Eur. J. Cancer (1965).

[28] Meng J., Peng J., Feng J., Maurer J., Li X., Li Y., Yao S., Chu R., Pan X., Li J. (2021). Niraparib exhibits a synergistic anti-tumor effect with PD-L1 blockade by inducing an immune response in ovarian cancer. J. Transl. Med..

[29] Kumar T., Hobbs E., Yang F., Chang J.T., Contreras A., Cuentas E.R.P., Garber H., Lee S., Lu Y., Scoggins M.E. (2022). Tumor Immune Microenvironment Changes by Multiplex Immunofluorescence Staining in a Pilot Study of Neoadjuvant Talazoparib for Early-Stage Breast Cancer Patients with a Hereditary BRCA Mutation. Clin. Cancer Res..

[30] Kohlhapp F.J., Haribhai D., Mathew R., Duggan R., Ellis P.A., Wang R., Lasater E.A., Shi Y., Dave N., Riehm J.J. (2021). Venetoclax Increases Intratumoral Effector T Cells and Antitumor Efficacy in Combination with Immune Checkpoint Blockade. Cancer Discov..

[31] Cai G., Karni A., Oliveira E.M., Weiner H.L., Hafler D.A., Freeman G.J. (2004). PD-1 ligands, negative regulators for activation of naive, memory, and recently activated human CD4+ T cells. Cell Immunol..

[32] Liu Z., Chu H., Zhao W., Yang C., Liu T., Shen N., Tang Z. (2025). Polymeric Multivalent Fc Binding Peptides-Fabricated Clinical Compounding Bispecific Antibody Potentiates Dual Immunotherapy Targeting PD1 and CTLA-4. Adv. Sci..

[33] Thibult M.L., Mamessier E., Gertner-Dardenne J., Pastor S., Just-Landi S., Xerri L., Chetaille B., Olive D. (2013). PD-1 is a novel regulator of human B-cell activation. Int. Immunol..

[34] Wang C., Zhao Y., Zhang S., Du M., He G., Tan S., Li H., Zhang D., Cheng L. (2024). Single-cell RNA sequencing reveals the heterogeneity of MYH11+ tumour-associated fibroblasts between left-sided and right-sided colorectal cancer. J. Cell. Mol. Med..

[35] Lei H., Huang L., Wan H., Chen M. (2025). Overexpression of LMOD1 induces oxidative stress and enhances cell apoptosis of melanoma through the RIG-I like receptor pathway. Biochim. Biophys. Acta Mol. Basis Dis..

[36] Wu S.Y., Zhang S.W., Ma D., Xiao Y., Liu Y., Chen L., Song X.Q., Ma X.Y., Xu Y., Chai W.J. (2023). CCL19(+) dendritic cells potentiate clinical benefit of anti-PD-(L)1 immunotherapy in triple-negative breast cancer. Med.

[37] Feng X., Wang Z., Cen M., Zheng Z., Wang B., Zhao Z., Zhong Z., Zou Y., Lv Q., Li S. (2025). Deciphering potential molecular mechanisms in clear cell renal cell carcinoma based on the ubiquitin-conjugating enzyme E2 related genes: Identifying UBE2C correlates to infiltration of regulatory T cells. Biofactors.

[38] Wang Z., Xie M., Jia Z., Tao Z., Zhao P., Ying M. (2024). FOXF1 inhibits invasion and metastasis of lung adenocarcinoma cells and enhances anti-tumor immunity via MFAP4/FAK signal axis. Sci. Rep..

[39] Wang X., Wang J., Shen H., Luo Z., Lu X. (2022). Downregulation of TPX2 impairs the antitumor activity of CD8+ T cells in hepatocellular carcinoma. Cell Death Dis..

[40] Wu J., Zhang L., Li W., Wang L., Jia Q., Shi F., Li K., Liao L., Shi Y., Wu S. (2023). The role of TOP2A in immunotherapy and vasculogenic mimicry in non-small cell lung cancer and its potential mechanism. Sci. Rep..

[41] Yoon S.J., Park J., Shin Y., Choi Y., Park S.W., Kang S.G., Son H.Y., Huh Y.M. (2020). Deconvolution of diffuse gastric cancer and the suppression of CD34 on the BALB/c nude mice model. BMC Cancer.

[42] Oh S.C., Sohn B.H., Cheong J.H., Kim S.B., Lee J.E., Park K.C., Lee S.H., Park J.L., Park Y.Y., Lee H.S. (2018). Clinical and genomic landscape of gastric cancer with a mesenchymal phenotype. Nat. Commun..

[43] Ooi C.H., Ivanova T., Wu J., Lee M., Tan I.B., Tao J., Ward L., Koo J.H., Gopalakrishnan V., Zhu Y. (2009). Oncogenic pathway combinations predict clinical prognosis in gastric cancer. PLoS Genet..

[44] Riaz N., Havel J.J., Makarov V., Desrichard A., Urba W.J., Sims J.S., Hodi F.S., Martín-Algarra S., Mandal R., Sharfman W.H. (2017). Tumor and Microenvironment Evolution during Immunotherapy with Nivolumab. Cell.

[45] Gide T.N., Quek C., Menzies A.M., Tasker A.T., Shang P., Holst J., Madore J., Lim S.Y., Velickovic R., Wongchenko M. (2019). Distinct Immune Cell Populations Define Response to Anti-PD-1 Monotherapy and Anti-PD-1/Anti-CTLA-4 Combined Therapy. Cancer Cell.

[46] Hugo W., Zaretsky J.M., Sun L., Song C., Moreno B.H., Hu-Lieskovan S., Berent-Maoz B., Pang J., Chmielowski B., Cherry G. (2016). Genomic and Transcriptomic Features of Response to Anti-PD-1 Therapy in Metastatic Melanoma. Cell.

[47] Nathanson T., Ahuja A., Rubinsteyn A., Aksoy B.A., Hellmann M.D., Miao D., Van Allen E., Merghoub T., Wolchok J.D., Snyder A. (2017). Somatic Mutations and Neoepitope Homology in Melanomas Treated with CTLA-4 Blockade. Cancer Immunol. Res..

[48] Bhattacharya S., Andorf S., Gomes L., Dunn P., Schaefer H., Pontius J., Berger P., Desborough V., Smith T., Campbell J. (2014). ImmPort: Disseminating data to the public for the future of immunology. Immunol. Res..

[49] Li Y., Jiang T., Zhou W., Li J., Li X., Wang Q., Jin X., Yin J., Chen L., Zhang Y. (2020). Pan-cancer characterization of immune-related lncRNAs identifies potential oncogenic biomarkers. Nat. Commun..

[50] Repana D., Nulsen J., Dressler L., Bortolomeazzi M., Venkata S.K., Tourna A., Yakovleva A., Palmieri T., Ciccarelli F.D. (2019). The Network of Cancer Genes (NCG): A comprehensive catalogue of known and candidate cancer genes from cancer sequencing screens. Genome Biol..

[51] Lu X., Meng J., Zhou Y., Jiang L., Yan F. (2021). MOVICS: An R package for multi-omics integration and visualization in cancer subtyping. Bioinformatics.

[52] Knijnenburg T.A., Wang L., Zimmermann M.T., Chambwe N., Gao G.F., Cherniack A.D., Fan H., Shen H., Way G.P., Greene C.S. (2018). Genomic and Molecular Landscape of DNA Damage Repair Deficiency across The Cancer Genome Atlas. Cell Rep..

[53] Yoshihara K., Shahmoradgoli M., Martínez E., Vegesna R., Kim H., Torres-Garcia W., Treviño V., Shen H., Laird P.W., Levine D.A. (2013). Inferring tumour purity and stromal and immune cell admixture from expression data. Nat. Commun..

[54] He Y., Jiang Z., Chen C., Wang X. (2018). Classification of triple-negative breast cancers based on Immunogenomic profiling. J. Exp. Clin. Cancer Res..

[55] Bagaev A., Kotlov N., Nomie K., Svekolkin V., Gafurov A., Isaeva O., Osokin N., Kozlov I., Frenkel F., Gancharova O. (2021). Conserved pan-cancer microenvironment subtypes predict response to immunotherapy. Cancer Cell.

[56] Hänzelmann S., Castelo R., Guinney J. (2013). GSVA: Gene set variation analysis for microarray and RNA-seq data. BMC Bioinform..

[57] Sanchez-Vega F., Mina M., Armenia J., Chatila W.K., Luna A., La K.C., Dimitriadoy S., Liu D.L., Kantheti H.S., Saghafinia S. (2018). Oncogenic Signaling Pathways in The Cancer Genome Atlas. Cell.

[58] Ritchie M.E., Phipson B., Wu D., Hu Y., Law C.W., Shi W., Smyth G.K. (2015). limma powers differential expression analyses for RNA-sequencing and microarray studies. Nucleic Acids Res..

[59] Yu G., Wang L.G., Han Y., He Q.Y. (2012). clusterProfiler: An R package for comparing biological themes among gene clusters. Omics J. Integr. Biol..

[60] Hoshida Y. (2010). Nearest template prediction: A single-sample-based flexible class prediction with confidence assessment. PLoS ONE.

[61] Wu J., Yao J., Jia S., Yao X., Shao J., Cao W., Ma S., Yao X., Li H. (2023). A cuproptosis-related lncRNA signature for predicting prognosis and immune response in hepatocellular carcinoma. Heliyon.

[62] Maeser D., Gruener R.F., Huang R.S. (2021). oncoPredict: An R package for predicting in vivo or cancer patient drug response and biomarkers from cell line screening data. Brief. Bioinform..

[63] Wishart D.S., Knox C., Guo A.C., Cheng D., Shrivastava S., Tzur D., Gautam B., Hassanali M. (2008). DrugBank: A knowledgebase for drugs, drug actions and drug targets. Nucleic Acids Res..

[64] Long J., Wang D., Wang A., Chen P., Lin Y., Bian J., Yang X., Zheng M., Zhang H., Zheng Y. (2022). A mutation-based gene set predicts survival benefit after immunotherapy across multiple cancers and reveals the immune response landscape. Genome Med..

[65] Liu H., Zhang W., Zhang Y., Adegboro A.A., Fasoranti D.O., Dai L., Pan Z., Liu H., Xiong Y., Li W. (2024). Mime: A flexible machine-learning framework to construct and visualize models for clinical characteristics prediction and feature selection. Comput. Struct. Biotechnol. J..

[66] Ayers M., Lunceford J., Nebozhyn M., Murphy E., Loboda A., Kaufman D.R., Albright A., Cheng J.D., Kang S.P., Shankaran V. (2017). IFN-γ-related mRNA profile predicts clinical response to PD-1 blockade. J. Clin. Investig..

[67] Topalian S.L., Hodi F.S., Brahmer J.R., Gettinger S.N., Smith D.C., McDermott D.F., Powderly J.D., Carvajal R.D., Sosman J.A., Atkins M.B. (2012). Safety, activity, and immune correlates of anti-PD-1 antibody in cancer. N. Engl. J. Med..

[68] Dominguez C.X., Müller S., Keerthivasan S., Koeppen H., Hung J., Gierke S., Breart B., Foreman O., Bainbridge T.W., Castiglioni A. (2020). Single-Cell RNA Sequencing Reveals Stromal Evolution into LRRC15(+) Myofibroblasts as a Determinant of Patient Response to Cancer Immunotherapy. Cancer Discov..

[69] Rooney M.S., Shukla S.A., Wu C.J., Getz G., Hacohen N. (2015). Molecular and genetic properties of tumors associated with local immune cytolytic activity. Cell.

[70] Jerby-Arnon L., Shah P., Cuoco M.S., Rodman C., Su M.J., Melms J.C., Leeson R., Kanodia A., Mei S., Lin J.R. (2018). A Cancer Cell Program Promotes T Cell Exclusion and Resistance to Checkpoint Blockade. Cell.

[71] Shukla S.A., Bachireddy P., Schilling B., Galonska C., Zhan Q., Bango C., Langer R., Lee P.C., Gusenleitner D., Keskin D.B. (2018). Cancer-Germline Antigen Expression Discriminates Clinical Outcome to CTLA-4 Blockade. Cell.

[72] Auslander N., Zhang G., Lee J.S., Frederick D.T., Miao B., Moll T., Tian T., Wei Z., Madan S., Sullivan R.J. (2018). Robust prediction of response to immune checkpoint blockade therapy in metastatic melanoma. Nat. Med..

[73] Cui C., Xu C., Yang W., Chi Z., Sheng X., Si L., Xie Y., Yu J., Wang S., Yu R. (2021). Ratio of the interferon-γ signature to the immunosuppression signature predicts anti-PD-1 therapy response in melanoma. npj Genom. Med..

