# Supplementary material for: Identification of Immune&Driver Molecular Subtypes Optimizes Immunotherapy Strategies for Gastric Cancer
Source: Int J Mol Sci. 2026 Jan 9;27(2):696. doi: 10.3390/ijms27020696 (PMC12841528; doi:10.3390/ijms27020696)
Supplement: Supplementary file 1 [file ijms-27-00696-s001.zip › ijms-4054085-supplementary.pdf]

Supplementary Figures

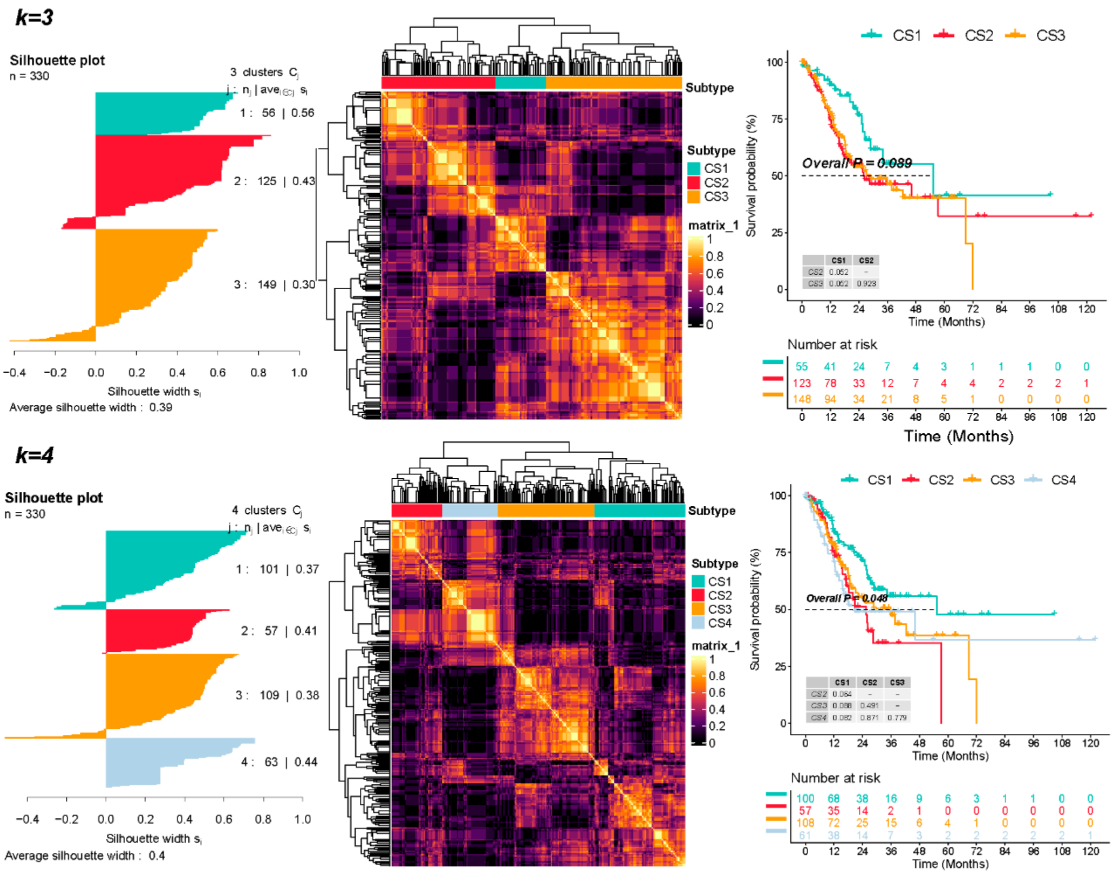

Figure S1. Integrative clustering analyses performed with k = 3 and k = 4.

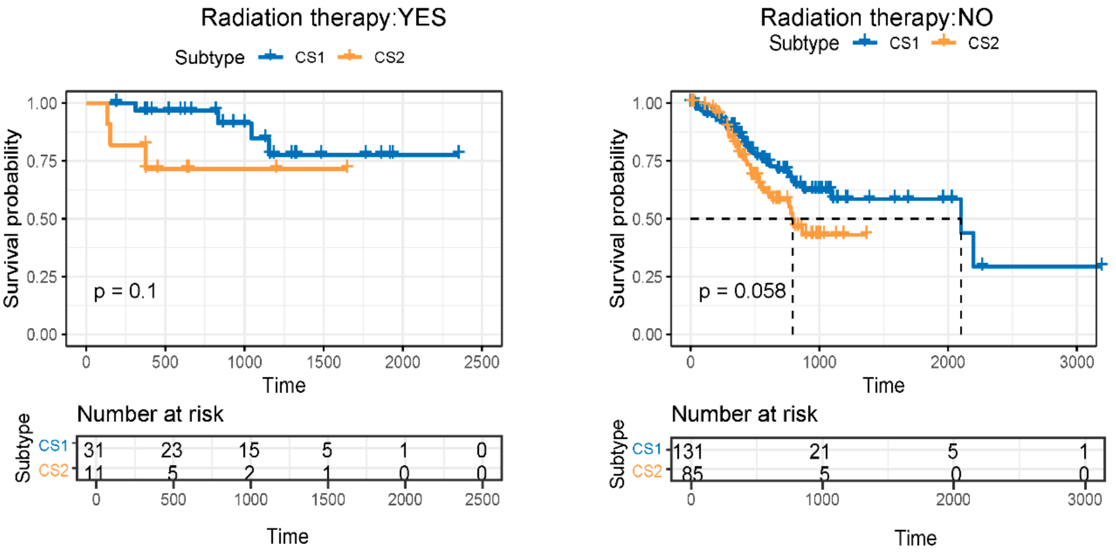

Figure S2. Overall Survival of CS1 and CS2 Stratified by Radiotherapy Status in the TCGA cohort.

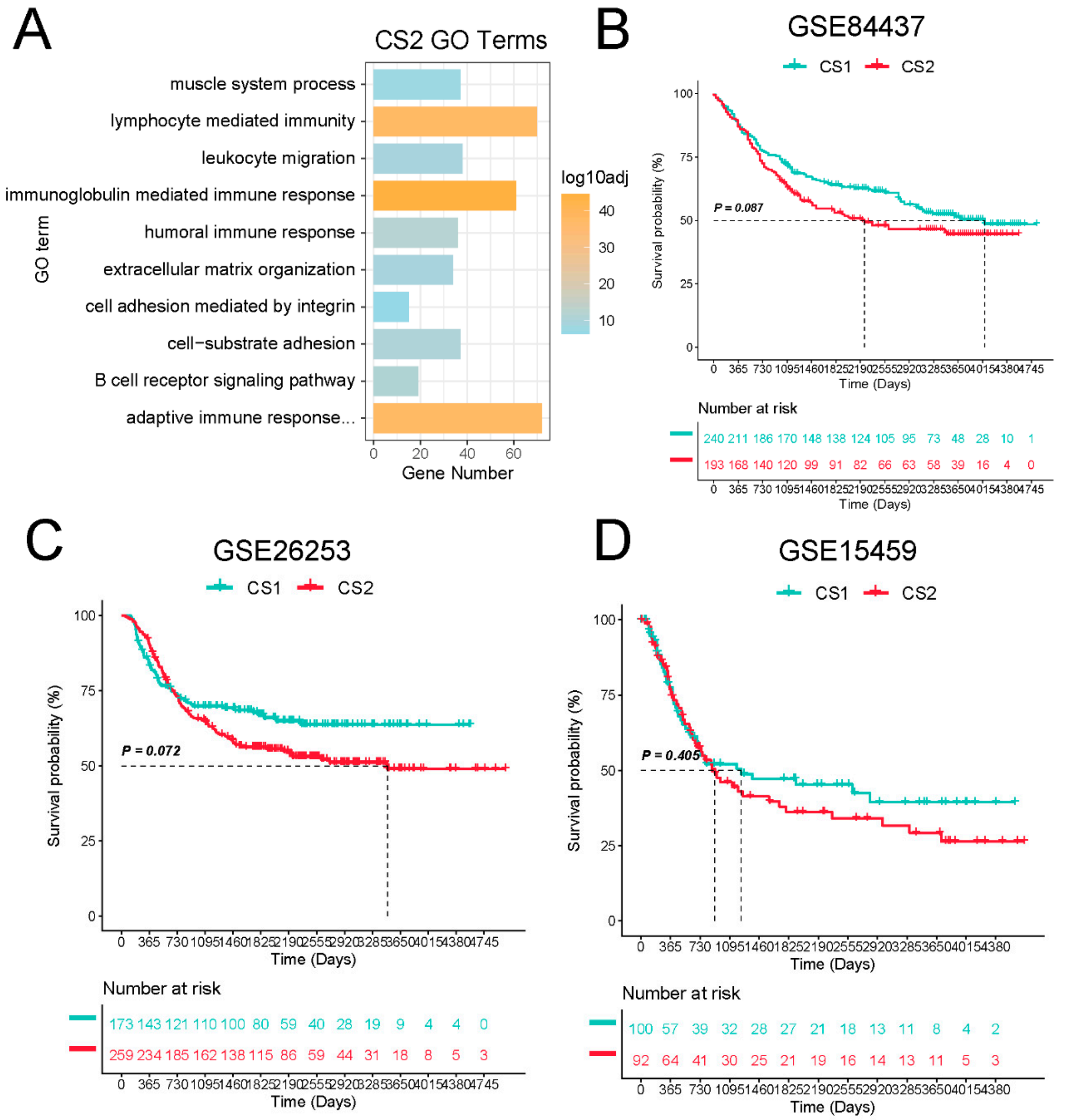

Figure S3. Comparative analyses using the full CS2 feature set. A. GO functional enrichment analysis of full biomarkers. B-D. Kaplan–Meier curves showing OS differences between subtypes across B. GSE84437, C. GSE26253, and D. GSE15459 cohorts.

Supplementary Tables

Table S1. Sample size and event distribution across training and validation cohorts.

dataset	N_total	DCB_Y_events	NDB_N_nonevents	Y_rate	features	EPV_events_per_feature	NPV_nonevents_per_feature
Training	42	24	18	0.571428571	1	24	18
Gide et al.-PD1	41	25	16	0.609756098	1	25	16
Gide et al.-PD1&CTLA4	32	26	6	0.8125	1	26	6
Nathanson et al.	15	4	11	0.266666667	1	4	11
Kim et al.	45	12	33	0.266666667	1	12	33

Table S2. Confusion and performance metrics of the PCA-based logistic regression model.

dataset	Accuracy	BalancedAcc	Sensitivity	Specificity	PPV	NPV	F1	Threshold
Training (OOF)	0.721428571	0.725694444	0.695833333	0.755555556	0.791469194	0.650717703	0.740576497	0.541429769
Gide et al.-PD1	0.487804878	0.4675	0.56	0.375	0.583333333	0.352941176	0.571428571	0.541429769
Gide et al.-PD1&CTLA4	0.53125	0.583333333	0.5	0.666666667	0.866666667	0.235294118	0.634146341	0.541429769
Nathanson et al.	0.533333333	0.602272727	0.75	0.454545455	0.333333333	0.833333333	0.461538462	0.541429769
Kim et al.	0.311111111	0.291666667	0.25	0.333333333	0.12	0.55	0.162162162	0.541429769
